# The relationship between young college students’ recognition of national COVID-19 crisis governance capabilities and the improvement of national identity: the mediating role of online participation in public health critical events

**DOI:** 10.3389/fpubh.2024.1349890

**Published:** 2024-05-15

**Authors:** Jun Xie, Na Zhu, Jia Tan, Hong Gao

**Affiliations:** ^1^Department of Student Affairs, University of South China, Hengyang, China; ^2^Department of Nursing, The Second Affiliated Hospital, Hengyang Medical School, University of South China, Hengyang, China; ^3^Department of Neonatology, The First Affiliated Hospital, Hengyang Medical School, University of South China, Hengyang, China

**Keywords:** young college students, mediation model, national COVID-19 crisis governance capabilities, national identity, online participation in public health critical event

## Abstract

**Background:**

Improving the young college students’ national identity is crucial for ensuring social stability and fostering development during public health critical events such as COVID-19. Young college students’ recognition of national COVID-19 crisis governance capabilities can influence their national identity, and online participation in public health criticalevents may serve as a crucial role in shaping this intricate relationship. To investigate this possibility, the present study established an intermediary model to examine the impact of online participation in public health critical events on young college students’ recognition of national COVID-19 crisis governance capabilities and improvement of national identity.

**Methods:**

This cross-sectional survey study employed a convenience sampling method to investigate a total of 3041 young college students in China. The correlations between study variables were analyzed using Spearman’s rank correlation. The mediation model was established using PROCESS Model 4 with 5000 bootstrap samples in SPSS. The bias-corrected bootstrap method provided statistical efficacy and identification interval estimation.

**Results:**

Young college students’ recognition of national COVID-19 crisis governance capabilities (*r*=0.729, *P*<0.001) and online participation in public health critical events (*r*=0.609, *P*<0.001) were positively correlated with improvement of their national identity. The relationship between these two factors was partially mediated by online participation in public health critical events (Indirect effect estimate=0.196, *P*<0.001).

**Conclusion:**

Online participation in public health critical events played a mediating role in the association between college students’ recognition of national COVID-19 crisis governance capabilities and the improvement of national identity. Our findings provide a novel intervention strategy for improving college students’ national identity, which is to encourage their online participation in public health critical events.

## Introduction

National identity is an individual’s subjective or internalized sense of belonging to their country (1). It is linked to the robustness of a nation’s internal cohesion and plays an indispensable role in the holistic advancement of its economy, culture, and politics. Public health critical events such as the COVID-19 epidemic were highly likely to impact individuals’ recognition, attitude, emotion, and belief, thereby influencing the construction of national identity. As a formidable driving force behind social progress, young college students constitute the primary agents in cultivating national identity. However, college students, being in the nascent stage of youth and possessing immature ideologies, are more susceptible to the influence of public health critical events on their national identity. Therefore, improving young college student’s national identity is an important cornerstone for maintaining social stability and fostering development during present or future crises. At present, there is a paucity of research focusing on improving college students’ national identity, particularly during the COVID-19 pandemic.

### The effect of young college students’ recognition of national COVID-19 crisis governance capabilities on their improvement of national identity

National governance capacity refers to the proficiency in effectively managing all aspects of social affairs through the utilization of national institutions (2). The prevention and control of the COVID-19 epidemic served as a significant litmus test for assessing the current national governance capacity, including crisis response capabilities, resource mobilization prowess, information dissemination capabilities, and efficacy in implementing preventive measures (3, 4).

According to the social cognition theory, social cognition pertains to an individual’s perception, cognitive processes, and comprehension of others as well as their social environment during the process of social interaction (5, 6). Young college students’ recognition of national COVID-19 crisis governance capabilities is a tangible manifestation of their social cognition in practical societal engagement. During the COVID-19 pandemic, a plethora of information proliferated across various online and offline platforms, making it arduous to discern veracity from falsehood (6). Consequently, certain inflammatory assertions inevitably arose and were directed toward the government. Young college students constitute the most proactive demographic in information acquisition. The potential impact of these inflammatory remarks on college students’ social cognition should not be underestimated. Studies have showed that an individual’s cognition of social phenomena significantly influences their sense of identity and belonging to society and the nation (7). During the prevention and control of COVID-19, the government’s dissemination of accurate and timely information played a pivotal role in effective communication, facilitating college students and all sectors of society to gain a comprehensive understanding of the current situation, as well as the array of emergency plans and prevention strategies implemented by the authorities (8, 9). This measure has gradually enhanced college students’ recognition of their contribution to national COVID-19 crisis governance capabilities. Following major public emergencies, the cognition of government emergency management ability and the level of social governance may exert a profound influence on emotional identification among college students (10). Young college students perceived that the inefficiency of government emergency management undermined their self-confidence and national pride, thereby eroding their national identity. On the contrary, the government’s efficient emergency management and social governance can effectively cater to young college student’s sense of collective superiority, thereby further enhancing their national identity (11). Hence, the young college students’ recognition of national crisis governance capabilities serves as a crucial foundation for enhancing national identity. Nevertheless, the internal mechanism between young college students’ recognition of national crisis governance capabilities and their national identity remains to be further verified.

### The effect of young college students’ recognition of national COVID-19 crisis governance capabilities on online participation in public health critical events

Amidst the COVID-19 pandemic, characterized by widespread implementation of social distancing measures, quarantine protocols, and isolation practices, social media has emerged as a considerable communication channel facilitating the reception and exchange of health-related information among individuals (12). During this special period, there has been a notable surge in social media usage across various demographics, with young college students emerging as the most actively engaged and frequently interacting group of readers (13).

Based on predict behavior and behavior change theory, social cognition has made important contributions in identifying determinants of health behavior and elucidating the underlying mechanisms involved (14). Young college students participated in activities related to major public health events through social media, and the change and adoption of these behaviors were promoted by cognitive activities such as risk perception and government crisis governance capabilities cognition (15). It was precisely because the government was fully aware of the significance of this aspect. Therefore, it strategically employed diverse online platforms to disseminate information and provide services, enabling young college students to fully comprehend the current epidemic prevention and control dynamics in a timely and effective manner, thereby potentially improving their recognition of national crisis governance capabilities (9, 16, 17). Building upon the recognition of national COVID-19 crisis governance capabilities, young college students exhibited a high level of acceptance toward policies and measures for COVID-19 prevention and control. Consequently, they channeled their enthusiasm into actively participating in online public health initiatives through various social media platforms. Online public health activities included following and disseminating epidemic-related information (such as policy issuance, crisis response measures, etc.), actively participating in anti-epidemic initiatives (including rescue operations, charity fundraising activities, etc.), and expressing individual opinions (such as discussing and sharing preventive measures with their relatives or friends) (18–20). From the perspective of reciprocal influences, the frequent utilization of social media had stimulated their active participation in public health critical events, while the process of discussion and interaction on social media had enhanced their recognition of national COVID-19 crisis governance capabilities. It can be inferred that the young college students’ recognition of national crisis governance capabilities may enhance their online participation in public health critical events, and these two factors may mutually influence each other.

### The effect of online participation in public health critical events on national identity

During the COVID-19 pandemic, online participation in public health critical events through social media had emerged as an important social activity. For instance, during the first half of 2020, the topic of mask-wearing had garnered extensive attention across various social media platforms. Young college students, serving as the primary driving force, actively participated in this process by using social media platforms to disseminate information regarding mask-wearing interventions through frequent posting, liking, commenting, reposting, and using hashtags to share their personal perspectives and experiences (20). In this way, they not only conveyed their individual attitudes and behaviors regarding mask-wearing but also fostered greater user engagement in discussions, thereby enhancing the effectiveness of publicity and educational initiatives. Moreover, young college students could utilize their professional expertise and competencies to provide assistance in various areas such as data entry, logistics support, remote education (18).

According to the emotions theory, emotions serve a clear evaluative function, expressing the association between recent events or outcomes and individual values (21). When individuals encountered unknown major public health events, their emotions often tended to be predominantly negative, thereby hindering the accurate assessment of the overall event. The social cognitive development of young college students was not flawless, and the impact of emotions on their social behavior and values should not be ignored. It could be reasonably inferred that the emotional response of college students to anti-epidemic behaviors was associated with their national identity. During the COVID-19 pandemic, effective and timely channeling of negative emotions among college students, coupled with their active engagement in anti-epidemic activities, could significantly enhance the efficacy of fostering a strong national identity. Scholars have confirmed this point that young college students exhibit a powerful sense of mission and strong sense of responsibility in anti-epidemic endeavors, thereby effectively stimulating their fervent patriotism, collective consciousness, and nationalistic pride (22). This further provided substantial material for their education in national identity. The more actively college students participated in epidemic prevention, the stronger their national identity became (11). Meanwhile, active participation in this process cultivated a sense of honor within individuals, leading to a profound recognition of the rights and privileges bestowed upon them as citizens. Consequently, it motivated them to steadfastly safeguard national interests and promote social stability. Therefore, facilitating the active participation of young college students in public health critical events through online platforms may be an important strategy for enhancing national identity.

Based on these theoretical models and previous empirical evidence, online participation in public health critical events may serve as a potential mechanism for young college students to effectively recognize the national COVID-19 crisis governance capabilities and improve their national identity. Therefore, we pose the following hypotheses:

*H1*: Young college students’ recognition of national COVID-19 crisis governance capabilities will be positively associated with online participation in public health critical events and the improvement of national identity.

*H2*: Online participation in public health critical events will be positively associated with the improvement of national identity.

*H3*: Online participation in public health critical events mediates the relationship between young college students’ recognition of national COVID-19 crisis governance capabilities and the improvement of national identity.

**Figure 1:**
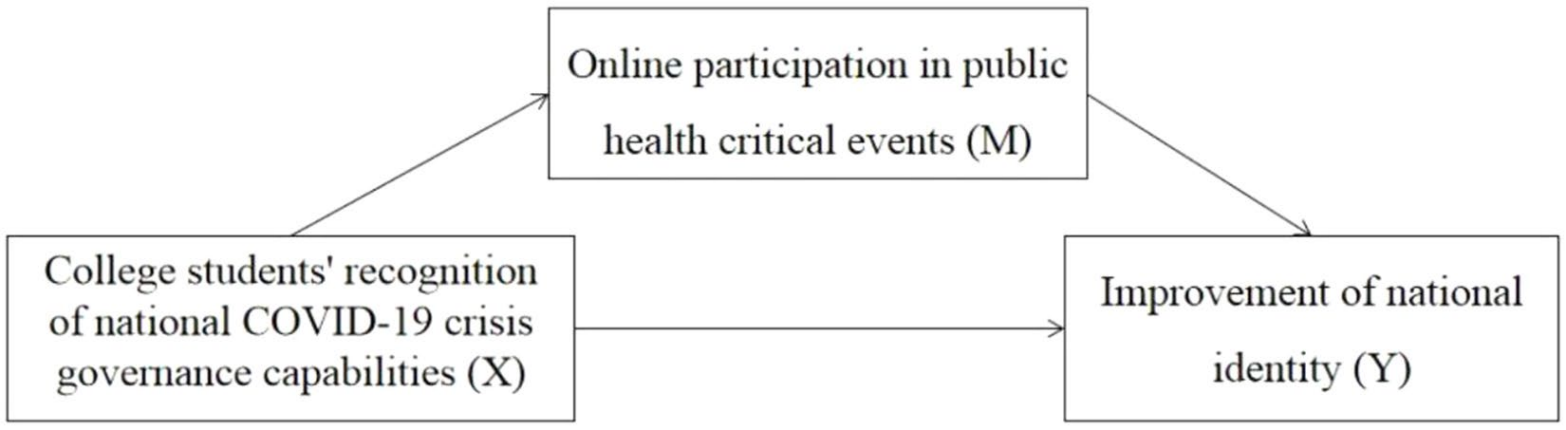
Research hypothesis framework of mediation model. A mediating model containing three pertinent variables hypothesized that online participation in public health critical events played a mediating role in the association between young college student’s recognition of national COVID-19 crisis governance capabilities and the improvement of national identity.

The above hypotheses are described in Figure 1.

## Methods

### Study design and participants

A cross-sectional study was conducted in three universities in Hunan Province of China from March 15 to April 19, 2020. Inclusion criteria were: (1) freshman, sophomore, junior, senior, and fifth year college students, (2) voluntarily participated in this online survey through the Wenjuanxing platform, and (3) provided informed consent. Exclusion criteria included participants who withdrew from the study without completing the questionnaire in its entirety. Demographic information in the self-report questionnaire included age, gender, nationality, university, residences, grade level, majors, etc. A total of 3,334 college students from three universities were enrolled in the study. The total response rate was 100%, including 3,041 valid questionnaires, with an effective rate of 91.2%. All participants provided informed consent before completing the questionnaire, and each participant was paid a total of 7 yuan ($1).

## Measurements

### Young college students’ recognition of national COVID-19 crisis governance capabilities

College students’ recognition of national COVID-19 crisis governance capabilities (RNCCC) was evaluated using eight items. Item 1: Do you think the national government has implemented bold and decisive measures? Item 2: Do you think state leaders attach great importance to the prevention and control of the epidemic? Item 3: Do you think the national government effectively organizes medical personnel to carry out the rescue? Item 4: Do you think the local government has implemented bold and decisive measures? Item 5: Do you think the state strongly publicizes knowledge of epidemic prevention and control through various media channels? Item 6: Do you think people all across the country have united to fight the epidemic? Item 7: Are you staying at home or reducing going out? Item 8: Do you think what is good for the country has been beneficial to you? Students responded were either “yes” (scored 1 point) or “no” (scored 0). All subjects could choose either “yes” or “no” based on their actual situation. The total score of this questionnaire is the sum of the scores for each item. Higher scores reflect a higher level of recognition among students regarding the national COVID-19 crisis governance capabilities. The questionnaire was developed by the research team based on their knowledge, combined with literature research, expert consultation, and survey tools (23–27). Cronbach’s alpha value of the scale was 0.807.

### Online participation in public health critical events

College students’ online participation in public health critical events (OPPHCE) was assessed using seven items. Item 1: I support and abide by the policies and measures taken by the national government online to prevent and control the COVID-19 pandemic. Item 2: I support and abide by the policies and measures taken by my local government online to prevent and control the COVID-19 pandemic. Item 3: I always follow the progress of the epidemic through online media. Item 4: I disseminate COVID-19 prevention knowledge through various internet platforms such as microblogs, Wechat, client, etc. Item 5: I discuss COVID-19 with friends and relatives online. Item 6: I take the initiative to participate in volunteer activities or serve as a staff member in charitable donation activities, such as transporting surgical masks, isolation devices, or food donated by the public to the epidemic area. Item 7: I support medical workers in conducting online epidemic prevention and control work. Each item is assessed using a 4-point scale. All subjects could choose the most suitable statement based on their actual situation. The highest score is 4 for “I participate in it every day” and the lowest score is 1 for “not relevant to me at all.” The total score is the sum of the scores for each item. Higher scores reflect a higher degree of online participation in public health critical events. The questionnaire was compiled by the research team based on their knowledge, combined with literature research, expert consultation, and survey tools (28–32). Cronbach’s alpha value of the scale was 0.921.

### Improvement of national identity

Seven items were used to assess the improvement of young college students’ national identity (INI). Item 1: Did your national identity improve when you participated in the COVID 19 response through online media? Item 2: Did your sense of national pride enhance when you participated in the COVID-19 response through online media? Item 3: Did your national safety awareness increase when you participated in the COVID-19 response through online media? Item 4: Has your patriotism been strengthened by participating in the COVID-19 response through online media? Item 5: Has your collective spirits been heightened by participating in the COVID-19 response through online media? Item 6: Have you shown your solidarity and friendship by participating in the COVID-19 response through online media? Item 7: Has your trust in medical staff been enhanced by participating in the COVID-19 response through online media? Each item was assessed using a 5-point scale. All subjects could choose the most suitable statement based on their actual situation. The highest score is 5 for “the most improvement,” and the lowest score is 1 for “decline.” The total score is the sum of the scores for each item. Higher scores reflect an improvement in students’ national identity. The questionnaire was compiled by the research team based on their knowledge, combined with literature research, expert consultation, and survey tools (33–35). Cronbach’s alpha value of the scale was 0.962.

### Statistical analysis

All data were managed and analyzed using statistical packages for the social sciences software (SPSS, version 26.0) and Excel (Microsoft Corp, Redmond, WA, United States). Descriptive statistics were applied to analyze the demographic data and all study variables. The correlations between study variables were analyzed using Spearman’s rank correlation. The mediation model was tested using PROCESS Model 4 with 5,000 bootstrap samples in SPSS. The bias-corrected bootstrap method provided statistical efficacy and identification interval estimation. College students’ recognition of national COVID-19 crisis governance capabilities was used as an independent variable, online participation in public health critical events was used as a mediate variable, and improvement of national identity was used as a dependent variable. The direct, indirect and total effects of the mediation model were considered to be statistically significant at the 0.05 probability level if the results with 95% bias-corrected identification interval (CI) did not include zero (36).

## Results

### Characteristics of the study population

A total of 3,334 college students participated in the study, of which 3,041 participants responded completely, resulting in a valid response rate of 91.2% (3041/3334). The mean age of the students was 19.06 ± 2.50 years. Among the surveyed college students, 51.3% were female, 85.7% were of Han nationality, and 25.4% lived in the cities (Table 1).

**Table 1:** Demographic characteristics of the study population (*N* = 3,041).

Variables	*n*	Percentage (%)
Gender		
Women	1,561	51.3
Men	1,480	48.7
Nationality		
Han	2,607	85.7
Other	434	14.3
Place of residence		
Cities	772	25.4
Towns	757	24.9
Villages	1,512	49.7
Grade		
Freshman	980	32.3
Sophomore	469	15.4
Junior	564	18.5
Senior	516	17.0
Fifth year	512	16.8
Subject category		
Human sciences	673	22.1
Natural sciences	522	17.2
Engineering	580	19.1
Medicine	1,266	41.6

### Descriptive statistics for the RNCCC, INI, and OPPHCE

The descriptive statistics for college students’ recognition of national COVID-19 crisis governance capabilities, improvement of national identity, and online participation in public health critical events are shown in Table 2. The average score for college students’ recognition of national COVID-19 crisis governance capabilities was 0.83 ± 0.34, and the score for the item “staying at home or reducing going out” was the highest. The average score for improvement of national identity was 4.13 ± 1.05, and the score for the item “sense of national pride enhance during participated in the COVID-19 response through online media” was the highest. The average score for online participation in public health critical events was 3.08 ± 0.73, and the score for the item “follow the progress of the epidemic through news reports” was the highest.

**Table 2:** Descriptive statistics for the RNCCC, INI, and OPPHCE.

Construct	Item	Mean	SD
College students’ recognition of national COVID-19 crisis governance capabilities (RNCCC) ^a^	RNCCC-1	0.78	0.41
	RNCCC-2	0.86	0.34
	RNCCC-3	0.87	0.34
	RNCCC-4	0.75	0.43
	RNCCC-5	0.69	0.46
	RNCCC-6	0.75	0.43
	RNCCC-7	1	0.05
	RNCCC-8	0.94	0.24
Improvement of national identity (INI) ^b^	INI-1	4.15	1.07
	INI-2	4.21	1.04
	INI-3	4.10	1.05
	INI-4	4.12	1.07
	INI-5	4.01	1.06
	INI-6	4.06	1.05
	INI-7	4.25	0.99
Online participation in public health critical events (OPPHCE) ^c^	OPPHCE-1	3.18	0.74
	OPPHCE-2	3.18	0.72
	OPPHCE-3	3.26	0.73
	OPPHCE-4	3.19	0.68
	OPPHCE-5	2.95	0.74
	OPPHCE-6	2.71	0.79
	OPPHCE-7	3.09	0.72

^a^2-point scale with a range of 1–2, ^b^5-point scale with a range of 1–5, ^c^4-point scale with a range of 1–4.

### Evaluation of the measurement tools

The construct reliability and validity of the measurement model were considered satisfactory (Table 3). According to Hair et al., the indicator loadings should reach a standard value of 0.70. Loadings between 0.40 and 0.70 should only be removed if their removal can improve the overall reliability to a minimum threshold (37). There were four indicator loadings ranging from 0.62 to 0.69, and the remaining indicator loadings exceeded 0.70. The removal and analysis of indicators with loadings less than 0.70 did not improve overall reliability. Therefore, all indicators were retained for this study. The results showed that the indicator loadings exhibited satisfactory levels of indicator reliability.

**Table 3:** Evaluation of measurement tools.

Construct	Item	Loadings	Cronbach’s alpha	Composite reliability	AVE*
College students’ recognition of national COVID-19 crisis governance capabilities (RNCCC)	RNCCC-1	0.707	0.807	0.885	0.502
	RNCCC-2	0.777			
	RNCCC-3	0.773			
	RNCCC-4	0.739			
	RNCCC-5	0.633			
	RNCCC-6	0.624			
	RNCCC-7	0.652			
	RNCCC-8	0.688			
Improvement of national identity (INI)	INI-1	0.768	0.962	0.933	0.667
	INI-2	0.792			
	INI-3	0.842			
	INI-4	0.853			
	INI-5	0.826			
	INI-6	0.855			
	INI-7	0.775			
Online participation in public health critical events (OPPHCE)	OPPHCE-1	0.822	0.921	0.939	0.687
	OPPHCE-2	0.886			
	OPPHCE-3	0.892			
	OPPHCE-4	0.883			
	OPPHCE-5	0.754			
	OPPHCE-6	0.727			
	OPPHCE-7	0.823			

^*^Average variance extracted.

### Spearman correlation

Bivariate correlations were analyzed using Spearman’s rank correlation method, as shown in Table 4. All study variables were significantly correlated with each other. Improvement of college students’ national identity was positively correlated with college students’ recognition of national COVID-19 crisis governance capabilities (*r* = 0.729, *p* < 0.001), and their online participation in public health critical events (*r* = 0.609, *p* < 0.001).

**Table 4:** Spearman correlations among study variables.

Variables	1	2	3
College students’ recognition of national COVID-19 crisis governance capabilities	1		
Improvement of national identity	0.729^**^	1	
Online participation in public health critical events	0.563^**^	0.609^**^	1
Mean	6.64	28.84	21.54
SD	1.79	6.61	4.23

*N* = 3,041. **All correlations were significant at *p* < 0 0.001 (two tailed).

### Mediation effect models of study variables

**Figure 2:**
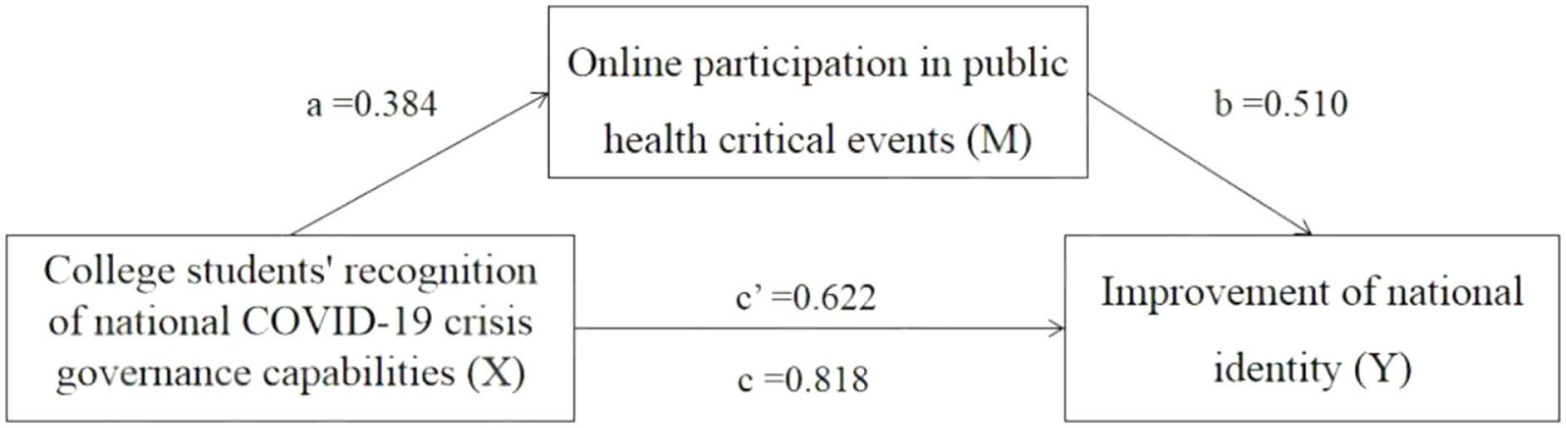
Mediation model examining the impact of young college students’ recognition of national COVID-19 crisis governance capabilities on their improvement of national identity through online participation in public health critical events. a = direct effect of X on mediator M; b = direct effect of mediator M on Y; c = total effect of X on Y; c’ = direct effect of X on Y. Mediation model with standardized coefficients. The positive direct effect coefficient of college student’s recognition of national COVID-19 crisis governance capabilities (X) on online participation in public health critical events (M) was 0.384. The positive direct effect coefficient of online participation in public health critical events (M) on the improvement of national identity (Y) was 0.510. The direct and total effect coefficients of college student’s recognition of national COVID-19 crisis governance capabilities (X) on the improvement of national identity (Y) were 0.622 and 0.818, respectively.

College students’ online participation in public health critical events was identified as a mediator between the recognition of national COVID-19 crisis governance capabilities and the improvement of national identity. Model 4 from PROCESS analysis was used to evaluate the mediation effect of the study variables. The structural model was statistically significant (*F =* 272.793, *p* < 0.001), and the *R* and *R^2^* values were 0.390 and 0.152, respectively (Table 5). The positive direct effect of the college students’ recognition of national COVID-19 crisis governance capabilities on the improvement of national identity was 0.622 (*p* < 0.001). The positive indirect effect of the college students’ recognition of national COVID-19 crisis governance capabilities (X) on the improvement of national identity (Y) through online participation in public health critical events (M) was 0.196 (*p* < 0.001), so the mediating effect of online participation in public health critical events was partial. The total effect of college students’ recognition of national COVID-19 crisis governance capabilities (X) on the improvement of national identity (Y) was 0.818 (*p* < 0.001). The results of direct effect, indirect effect and total effect are shown in Table 6. The mediation effect accounted for 24.0% of the total effect. The mediation effect accounted for 31.5% of the direct effect. Therefore, these results suggested that there was a partial mediating effect. Figure 2 shows the mediating effect structure model.

**Table 5:** Regression model with improvement of national identity as outcome variable.

Variables	coeff	se	*t* value	*p* value	LLCI	ULCI
Constant	13.719	0.659	20.823	<0.001	12.427	15.010
Online participation in public health critical events	0.510	0.026	19.253	<0.001	0.458	0.562
College students’ recognition of national COVID-19 crisis governance capabilities	0.622	0.063	9.921	<0.001	0.499	0.744

**Table 6:** Total, direct, and indirect effects of mediation analysis.

Effect	Estimate	*t/Z* value	*P* value	95% LCL	95% UCL
Total effect	0.818	12.488	<0.001	0.689	0.946
Direct effect	0.622	9.921	<0.001	0.499	0.744
Indirect effect	0.196	8.204*	<0.001	0.144	0.253

^*^Z value.

## Discussion

In this study, we first evaluated the role of online participation in public health critical events in young college students’ recognition of national COVID-19 crisis governance capabilities and improvement of national identity. We found that the improvement of national identity was positively correlated with college student’s recognition of national COVID-19 crisis governance capabilities and online participation in public health critical events. According to the model, online participation in public health critical events played a mediating role in the association between college students’ recognition of national COVID-19 crisis governance capabilities and the improvement of national identity.

Our study found that the college students’ recognition of national crisis governance capabilities was positively associated with the improvement of their national identity. During the prevention and control of COVID-19, the government has progressively deepened college students’ recognition of national crisis governance capabilities through public announcements, implementation of national crisis prevention and control measures, and media coverage. Our study revealed that college students generally recognized the effectiveness of the government’s epidemic prevention and control measures, and maintained a positive recognition of the government’s governance capabilities. Liu et al. (38) demonstrated that college students were satisfied with the national governance system and its capabilities during the pandemic. Approximately 90% of college students expressed that the performance of the national governance crisis had exceeded their initial expectations (38). College students’ satisfaction with epidemic prevention and control measures was positively correlated with their positive emotions (39).

The findings of this study suggested that the effectiveness of government governance significantly enhanced their national identity, pride and self-confidence. In particular, young college students had the highest average score (4.25 ± 0.99) for feeling a greater sense of pride in the government’s achievements in effectively controlling the epidemic. Furthermore, the achievement of epidemic prevention had strengthened their confidence and trust in medical staff. The effective national control of the COVID-19 pandemic had instilled great confidence in 89% of college students amidst these challenging times. Najima et al. found that 96.60% of college students felt more secure and confident (1). Therefore, the positive recognition of college students’ toward the national COVID-19 crisis governance capabilities was an effective strategy to enhance their national identity.

This study demonstrated that online participation in public health critical events played a partially mediating role in enhancing college students’ recognition of national COVID-19 crisis governance capabilities and improving national identity. During the pandemic period, the nation effectively utilized online media to facilitate the dissemination of prevention and control information as well as the implementation of measures, thereby enhancing young college students’ comprehension of the national COVID-19 crisis governance capabilities. College students extensively acquired and exchanged information through official WeChat accounts, official microblog pages, government websites, news outlets, as well as certain social and entertainment applications (9, 16, 17, 40). College students who possessed a greater amount of COVID-19 information were more motivated to take preventive measures (41, 42). According to the survey, a majority of students generally complied with government prevention requirements, actively disseminated knowledge on epidemic prevention and control online, and volunteered for charitable activities. Their active participation in epidemic prevention and control work had achieved remarkable outcomes, significantly enhancing their sense of pride and patriotism (43). Engaging in volunteer work has resulted in increased patriotism for 86% of volunteers, deeper national security awareness for 81%, and strengthened national unity awareness for 82%. Some studies indicated that with the continuous advancement of epidemic prevention and control work, college students would be more actively involved in the fight against the epidemic (44), thereby further enhancing their national unity and identity. It can be seen that college students’ recognition of national crisis governance capabilities encourages them to actively participate in COVID-19 prevention and control online, thereby improving their national identity. Simultaneously, the higher their recognition of national crisis governance capabilities, the stronger their sense of national identity, thus establishing a positive feedback loop. This positive feedback loop not only strengthened the national governance system, but also established a robust foundation for addressing similar challenges in the future.

The present study revealed the significant value of college students’ active participation in public health events online. Young college students were adhering to the government’s policy of staying at home in order to fight the highly contagious COVID-19 outbreak. During the epidemic period, the utilization of advanced online media platforms, government information disclosure, and a comprehensive mechanism for expressing citizens’ opinions provided practical conditions for college students to participate in public health events online from their homes. The active participation of college students in online public health events was crucial for controlling the spread of the epidemic, concurrently enhancing their sense of social responsibility, reducing psychological problems such as anxiety and depression, promoting self-identity, and further enhancing national identity (45). In view of this, the government should further increase dedicated funding for social practice, encourage college students to actively participate in public health events, guide them to consciously integrate personal development into social development, and internalize a sense of national belonging.

College students are in a formative stage of ideological culture and value orientation; their social cognitive ability, independent thinking ability, and moral discernment have not yet fully developed (46). The real-time reporting on the progress of COVID-19 prevention and control by network media has been the main channel for college students to understand governance capabilities. During this special period, the formation and rapid spread of false or negative news may cause panic, confusion, and social unrest among the public, thereby posing a great threat to national identity (47). Therefore, the government should guide accurate reporting on social media, enhance the dissemination of scientific knowledge, promote information disclosure, strengthen public participation, and guide the public to pay attention to international public opinion’s high recognition and evaluation of the government’s prevention and control measures, so as to create a social environment conducive to improving college students’ national identity (46, 48). Furthermore, it was crucial for governments to foster media literacy education in universities, including the assessment of online content credibility, management of information overload, and verification of information across various media platforms (49). This will help provide society as a whole with the necessary information literacy skills to effectively respond to future public health events.

### Strengths, limitations, and future research

This study first confirmed the research hypothesis that online participation in public health critical events could mediate the relationship between the college students’ recognition of national crisis governance capabilities and the improvement of their national identity. This study has a large sample size, ensuring strong representativeness and reliable results. These findings contribute to the advancement of theory and practice in national identity education among young college students.

Our study has three limitations. Firstly, this study employed college students’ self-reports to assess improvements in their national identity, potentially introducing response bias. Secondly, college students’ national identity was the result of the interaction of many factors. We only considered their online participation in public health critical events as a mediating factor. Finally, since mediating effects imply causation, the utilization of cross-sectional data for in-depth verification of mediating effects may not yield sufficiently convincing results. Therefore, it is imperative to conduct a longitudinal study to verify the mediating effect and further investigate the impact of individual, social, and other factors on predicting college students’ national identity. Besides, further research is required to determine their relevance and role in other types of public health critical events.

## Conclusion

In conclusion, this study first confirmed the positive association among college students’ recognition of national COVID-19 crisis governance capabilities, and the improvement of national identity, and online participation in public health critical events. Online participation in public health critical events played a mediating role in the association between college students’ recognition of national COVID-19 crisis governance capabilities and the improvement of national identity. Our findings provide a novel intervention strategy for improving college students’ national identity, which is to encourage their online participation in public health critical events.

## Data availability statement

The original contributions presented in the study are included in the article/supplementary material, further inquiries can be directed to the corresponding author.

## Ethics statement

The studies involving humans were approved by University of South China Medical Ethics Committee. The studies were conducted in accordance with the local legislation and institutional requirements. The participants provided their written informed consent to participate in this study.

## Author contributions

JX: Writing – original draft. NZ: Writing – original draft. JT: Writing – original draft. HG: Writing – review & editing.
